# Management of Patients with Inflammatory Diseases during the COVID-19 Pandemic

**DOI:** 10.31138/mjr.31.3.295

**Published:** 2020-09-22

**Authors:** Theofanis Apostolou, Ioannis E. Koutroubakis, Spilios Manolakopoulos, Gerasimos Mantzaris, Dimitrios Rigopoulos, Konstantinos Triantafyllou, Dimitrios Vassilopoulos

**Affiliations:** 1 President of the Hellenic Nephrological Society (HNS); 2 President and Board Member of the Hellenic Study Group for Inflammatory Bowel, Diseases (EOMIFNE); 3 President of the Hellenic Society for the Study of Liver (HASL); 4 President of the Hellenic Society of Dermatology and Venereology (HSDV); 5 President of the Hellenic Society of Gastroenterology (HSG); 6 President of the Greek Rheumatology Society & Professional Association of Rheumatologists (EPE-EPERE)

## Abstract

Patients with various inflammatory diseases of the gastrointestinal tract, skin, liver, kidneys, and musculoskeletal system-connective tissues, often undergo different anti-inflammatory therapies to maintain remission and avoid serious and/or life-threatening complications. Available data so far show an increased rate of hospitalization in such patients during the COVID19 pandemic. The key points of our position statement are summarized below:

- Patients with inflammatory diseases who receive moderate or high-risk anti-inflammatory therapies might be considered as an increased risk group for severe COVID-19 and appropriate measures should be taken in order to protect them.
- Initiation of immuno-suppressive/modulatory therapies should be done with caution, taking into account the severity of the underlying inflammatory disease, the type of anti-inflammatory treatment, and the risk of exposure to the SARS-CoV-2 virus.
- Discontinuation of anti-inflammatory therapies in patients who have not been exposed to or infected with the SARS-CoV-2 virus is not recommended.
- In patients who become infected with SARS-CoV-2, anti-inflammatory therapies should be discontinued, except in special cases.
- Specialty physicians should actively participate in the Interdisciplinary Teams caring for patients with inflammatory diseases during COVID19 infection.

## PURPOSE OF THIS POSITION STATEMENT

The pandemic of the novel SARSCoV-2 coronavirus has created a number of practical day-to-day problems in patients with inflammatory diseases undergoing treatment with different anti-inflammatory agents. These include patients with inflammatory diseases of the gastrointestinal tract (inflammatory bowel disease, IBD), liver (autoimmune hepatitis), skin (psoriasis, blistering skin disorders), kidneys (glomerulonephritis, glomerulopathies), and musculoskeletal-connective tissues diseases (rheumatoid arthritis, systemic rheumatic diseases such as Systemic Lupus Erythematosus, vasculitis, myositis, scleroderma, Sjögren’s Syndrome, etc.).

The purpose of this joint position statement is to provide up-to-date guidance for better management of patients with inflammatory diseases during the COVID19 pandemic. This text does not include recommendations for transplanted patients (kidney, liver) for whom there are special instructions from the Greek National Organization of Public Health (EODY).^1^

For the development of this joint position statement, the general recommendations of EODY^1^ as well as the most recent Specific Recommendations/Guidelines of the respective Greek^2–4^ and International^5–15^ Scientific Societies were taken into account. These statements do not replace the individual diagnostic and therapeutic decisions of treating physicians, and may be modified over time with the emergence of new scientific data.

## A. GENERAL STATEMENTS

1. Patients with inflammatory diseases (whether receiving immuno-suppressive/modulatory therapies or not) should follow the updated Guidelines and Recommendations of the National Organization of Public Health (EODY).
2. Up to date, there is no data to suggest that patients with inflammatory diseases are at higher risk of COVID-19 infection compared to the general population.
3. Although data on the course of COVID19 disease in patients with inflammatory diseases are still limited, recent findings from international databases of patients with IBD^16^ and rheumatic diseases^17^ show an increased hospitalization rate of these patients (31–46%), especially those receiving glucocorticoids. Based on these data and the recent Guidelines/Recommendations of International Health Organizations^18–19^ and Academic Hospitals,^20–21^ patients with inflammatory diseases undergoing moderate to high risk anti-inflammatory treatment (see Annex ) might be considered as an increased risk group for severe COVID-19 and appropriate measures should be taken in order to protect them.
4. Most specifically, regarding:
  - The home isolation of working patients and/or
  - The continuation, temporary or permanent discontinuation of their therapies, the following factors should be taken into account:
    - the severity of the underlying inflammatory disease (mild, moderate, severe, vital organ- and/or life-threatening) - patients’ comorbidities (age> 65 years, chronic respiratory or cardiovascular disease, chronic kidney or liver disease, diabetes mellitus)
    - the risk of exposure to SARS-CoV-2 (working-home environment, administration of IV therapies in a hospital setting)
    - the type of their anti-inflammatory therapies (see C and D, Annex)
5. In the Interdisciplinary Teams caring for patients with inflammatory diseases (who receive or not immuno-suppressive/modulatory therapies) who develop COVID19 infection, the inclusion of the respective specialists (gastroenterologists, dermatologists, hepatologists, nephrologists, rheumatologists) is mandatory.

## B. INITIATING THERAPY IN PATIENTS WITH ACTIVE INFLAMMATORY DISEASES WHO HAVE NOT BEEN EXPOSED TO OR INFECTED WITH SARS-CoV-2 VIRUS

1. For patients with active disease, the choice of initiating anti-inflammatory therapy should be based primarily on the severity of the underlying disease (mild, moderate, severe, vital organ- and/or life-threatening)
2. Therapeutic decisions should take into account:
  - the type of treatment (low vs. moderate - high risk, see Annex) and
  - the risk of exposure to SARS-CoV-2 in patients receiving such treatment (IV treatment in a hospital setting vs. pos/subcutaneous treatment at home, potential virus exposure at work or home)

## C. THERAPY OF PATIENTS WHO HAVE NOT BEEN EXPOSED TO OR INFECTED WITH SARS-CoV-2 VIRUS

1. **Glucocorticoids**
  Glucocorticoids should not be discontinued abruptly but caution must be taken to taper their dose to the lowest possible one that achieves adequate control of the underlying inflammatory disease.
2. Non-biologics/biologics/targeted-synthetic agents
  In the absence of SARS-CoV-2 exposure or COVID-19 infection, discontinuation of non-biologics, biologics or targeted-synthetic agents (see Annex) is not recommended.

## D. THERAPY OF PATIENTS WHO HAVE BEEN EXPOSED TO OR INFECTED WITH SARS-CoV-2 VIRUS

1. **Glucocorticoids**
  - It is recommended to taper glucocorticoids to the minimum possible dose required to control the inflammatory activity of the underlying disease.
  - In special cases, following a decision from the Interdisciplinary Team, glucocorticoids can be administered as treatment for the COVID19 infection.
  - In patients with mild to moderate IBD, conventional glucocorticoids may be substituted by budesonide (controlled ileal-release or multimatrix extended-release) to control the underlying inflammatory disease.
2. **Non-biologics**
  - It is recommended to stop or postpone their administration until patients are asymptomatic from COVID19 infection and the molecular viral tests become negative.
  - An exception is the administration of 5- Aminosalicylic acid (5-ASA, in patients with IBD) to control the underlying inflammatory disease which can be used safely, if necessary, at the maximum permitted dose and in all forms (orally and/or rectally).
  - In special cases, following a decision of the Interdisciplinary Team, hydroxychloroquine (in patients with systemic lupus erythematosus or rheumatoid arthritis) could be continued.
3. **Biologics/targeted-synthetic agents**
  - It is recommended to stop or postpone their administration until patients are asymptomatic from COVID19 infection and the molecular viral tests become negative.
  - In special cases, following a decision of the Interdisciplinary Team, the following agents may be continued:
    - Vedolizumab (in patients with active IBD) to control the underlying inflammatory disease and if necessary
    - Tocilizumab, for the control of their underlying inflammatory disease (in patients with rheumatoid arthritis or giant cell arteritis) and/or the treatment of COVID19 disease (according to existing National Guidelines for the treatment of COVID-19).

## ANNEX Risk categorization of anti-inflammatory therapies for severe COVID19

**Table:**

**ANTI-INFLAMMATORY THERAPIES**
**Low Risk**
**Glucocorticoids**
Budenoside (pos/rectally)
**Non-biologics**
Colchicine
Hydroxychloroquine (HCQ)
5-Aminosalicylic acid (5-ASA)
Sulfasalazine (SSZ)
**Biologics**
Anti-integrin (Vedolizumab)
**Moderate-High Risk**
**Glucocorticoids**
Prednisolone - Methylprednisolone (pos/IV)
**Non-biologics**
Azathioprine (AZA)
Cyclophosphamide (CYC)
Cyclosporine (CsA)
Leflunomide (LEF)
Methotrexate (MTX)
6-mercaptopurine (6-MP)
Mycophenolate acid (MPA)
Mycophenolate mofetil (MMF)
Tacrolimus
**Biologics**
Abatacept
Anti-IL1 (Anakinra, Canakinumab)
Anti-IL5 (Mepolizumab)
Anti-IL6 (Tocilizumab)
Anti-IL12/23 (Ustekinumab)
Anti-IL17 (Brodalumab, Secukinumab)
Anti-TNFs (Adalimumab, Certolizumab pegol, Etanercept, Golimumab, Infliximab)
Belimumab
Anti- B cell (Rituximab)
**Targeted synthetic agents**
Apremilast
JAK Inhibitors (Tofacitinib, Baricitinib)
